# Accessory Ossicles of the Foot and Ankle: Disorders and a Review of the Literature

**DOI:** 10.7759/cureus.1881

**Published:** 2017-11-26

**Authors:** Nigar Keles-Celik, Ozkan Kose, Rahime Sekerci, Gunes Aytac, Adil Turan, Ferhat Güler

**Affiliations:** 1 Department of Anatomy, Akdeniz University Faculty of Medicine; 2 Department of Orthopaedics and Traumatology, University of Health Sciences, Medical Faculty, Antalya Education and Research Hospital, Antalya, Turkey

**Keywords:** accessory ossicles, accessory navicular bone, os peroneum, os trigonum, os vesalianum

## Abstract

Accessory ossicles of the foot and ankle are normal variants of bone development that usually remain asymptomatic. However, they may be involved in various disorders and become a source of pain such as in fractures, dislocations, degenerative changes, osteonecrosis, osteoarthritis, osteochondral lesions, avascular necrosis, and irritation or impingement of adjacent soft tissues. Hence, during the assessment of the situations above, knowledge about these little-known ossicles could be very important to reach the correct diagnosis. Recent studies in the literature have mostly focused on the most frequent 9-12 accessory bones. In this review, 24 types of accessory ossicle are described. These ossicles are accessory navicular bone, os peroneum, os trigonum, os intermetatarseum, os vesalianum. os subfibulare, os subtibiale, os calcaneus secundarius, os calcanei accessorium, os supratalare, os sustentaculi, os talotibiale, os tali accessorium, talus secundarius, os subcalcis, os cuboideum secundarium, os supranaviculare, os infranaviculare, os paracuneiforme, os intercuneiforme, os cuneometatarsale I tibiale, os cuneometatarsale plantare, os cuneo–I metatarsale-II dorsale, and os aponeurosis plantaris. The clinical importance of these bones should be known thoroughly to reduce unnecessary orthopedic consultations and misdiagnosis. This article describes the clinical importance of the accessory ossicles and their possible pathological conditions. Understanding the possible disorders of the accessory ossicles of the foot and ankle can provide a more accurate diagnostic process.

## Introduction and background

**Figure 1:**
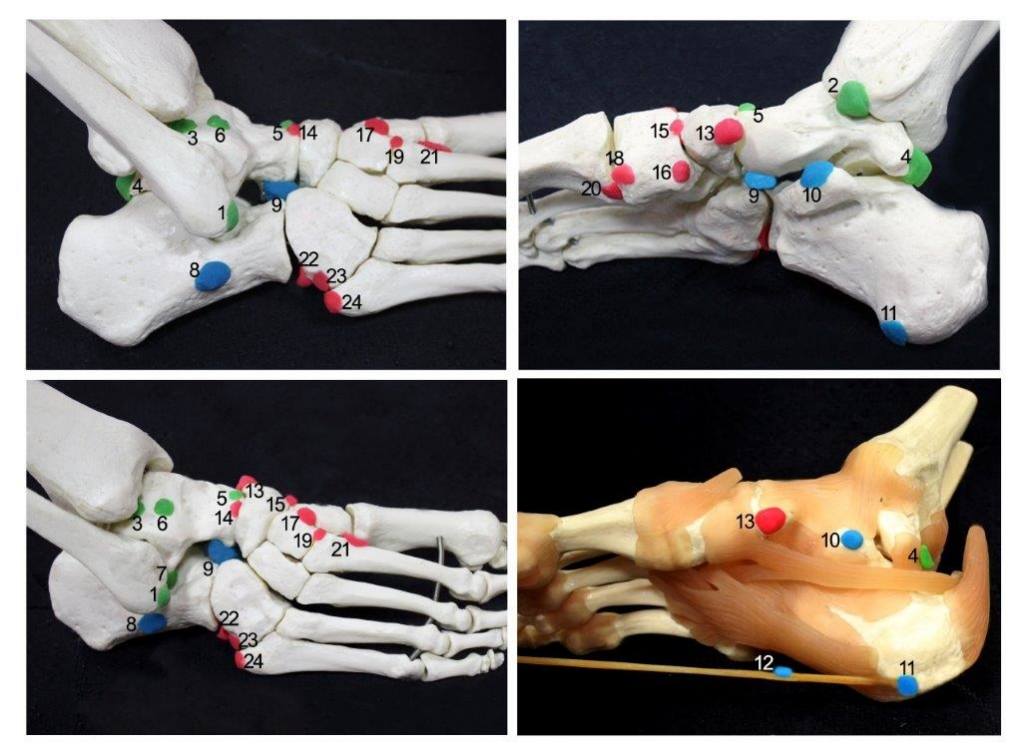
Schematic illustration showing the accessory ossicles defined regionally 1 - os subfibulare; 2 - os subtibiale; 3 - os talotibiale; 4 - os trigonum; 5 - os supratalare; 6 - os talus accessories; 7 - os talus secundarius; 8 - os calcanei accessorium; 9 - os calcanei secundarium; 10 - os sustentaculi; 11 - os subcalcis; 12 - os aponeurosis plantaris; 13 - accessory navicular bone; 14 - os supranaviculare ; 15 - os infranaviculare; 16 - os paracuneiforme; 17 - os intercuneiforme; 18 - os cuneometatarsale I tibiale; 19 - os cuneo–I metatarsale-II dorsale; 20 - os cuneometatarsale plantare; 21 - os intermetatarseum; 22 - os peroneum; 23 - os cuboideum secundarium; 24 - os vesalianum.

Accessory ossicles around the foot and ankle are common skeletal variations. They are usually derived from the failure of union of secondary ossification centers adjacent to the main bony mass. They might be either adjacent to the main bone or separate. These ossicles are mostly detected incidentally and might be unilateral or bilateral. There are various skeletal variations of the foot and ankle, including different accessory ossicles, bipartitions, and coalitions [1]. Numerous accessory ossicles of the foot and ankle are described in the current literature. Some of these bones are under-recognized accessory ossicles; therefore, different authors have named these bones differently, which causes confusion in the literature. For e.g., the accessory navicular bone is also known as tibiale externum, prehallux, os supranavicular, talonaviculare ossicle, and Pirie’s bone [2]. Accessory ossicles usually remain asymptomatic, but can become painful due to fractures, dislocations, degenerative changes, osteonecrosis, osteoarthritis, osteochondrial lesions, avascular necrosis, tumors, and irritation or impingement of adjacent soft tissues. Fractures (acute and stress) and dislocations are the most commonly reported causes of accessory ossicle disorders [3-5]. They are often confused with avulsion fractures. As a result of fractures, these bones may be infected or dislocated [1-2,6-9]. They may also simulate fractures and restrict the range of motion [2,6,10]. More recently, computed tomography (CT) and magnetic resonance imaging (MRI) scans have provided a clear understanding of their clinical relevance and helped to distinguish them from fractures [9]. Summers emphasized that accessory ossicles can be confused with fractures [11]. Therefore, the interpretation of X-rays could be difficult for emergency practitioners. It has been suggested that physicians should be confident in recognizing these ossicles and they should relate the patient’s clinical findings, symptoms, and past medical history with the concerned areas that are seen on the X-rays [12]. Recent studies have reported 9-12 accessory bones (Figure 1) [9,11,13].

A thorough knowledge of all accessory ossicles and their clinical significance is important to reduce unnecessary orthopedic consultations and misdiagnosis [14]. Therefore, in the present review, 24 types of accessory ossicle are described.

## Review

**Figure 2:**
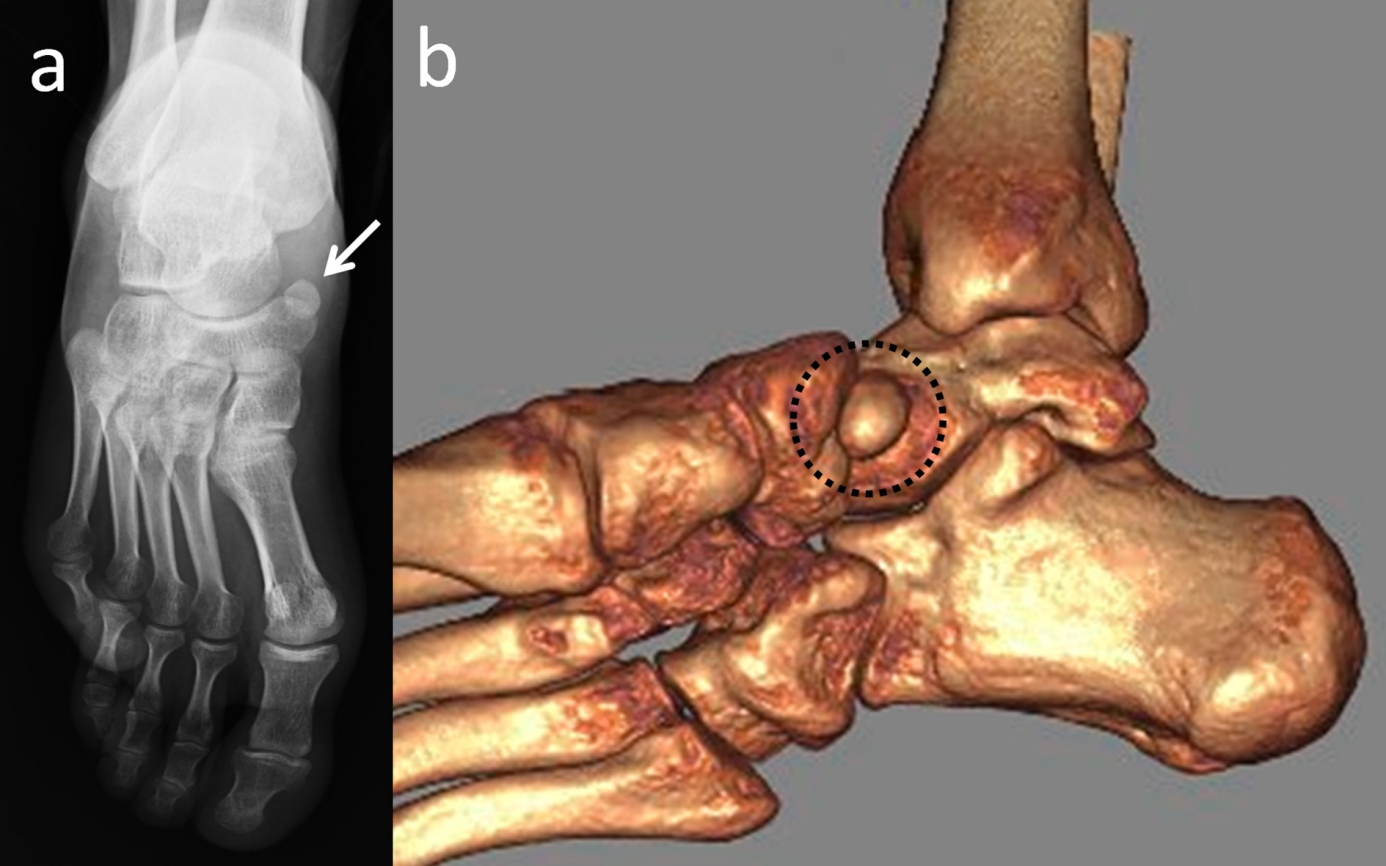
A 47-year-old male patient with an asymptomatic accessory navicular bone (a) Anteroposterior foot radiograph; (b) 3D computed tomography (CT) appearance of the inferomedial view.

The accessory navicular boneis also known as os naviculare secundarium, os tibiale externum, prehallux, and os scaphoideum accessorium*.* The incidence of the accessory navicular bone has been reported to be 4%-21% [9-10,15]. Three types of accessory navicular bones have been described. Type I is a 2.6-mm round shape within the posterior tibialis tendon, located up to 5 mm proximal to the navicular tuberosity (Figure 2).

This bone is also named as the os tibiale externum and naviculare secundarium. Type II originates from the secondary ossification center of the navicular bone and this type is also referred to as 'prehallux or bifurcate hallux’. Type II accessory ossicles are triangular or heart-shaped and its base is situated 1.2 mm from the medial and posterior aspects of the navicular bone. It is connected to the navicular tuberosity by a fibrocartilage or a hyaline cartilage layer. The accessory navicular may unite with the navicular tuberosity, thus forming the type, and is also known as the cornuate navicular bone. The cornuate navicular bone may occasionally be associated with painful conditions, adventitial bursa formation, or flatfoot deformity [9,16]. Type II accessory navicular bone symptoms are mostly seen because of traumatic or arthritic changes of the synchondrosis [9]. The symptoms are exacerbated during exercise or walking, affecting the sporting performance of adolescent athletes [17]. The accessory navicular bone usually co-exists with pes planus [2]. These bones should be differentiated from degenerative arthritic spurs, avulsion fractures, or traumatic conditions. When acute pain develops in this region, a bone scan may help to differentiate one of these accessory ossicles from an acute injury [10-11].

**Figure 3:**
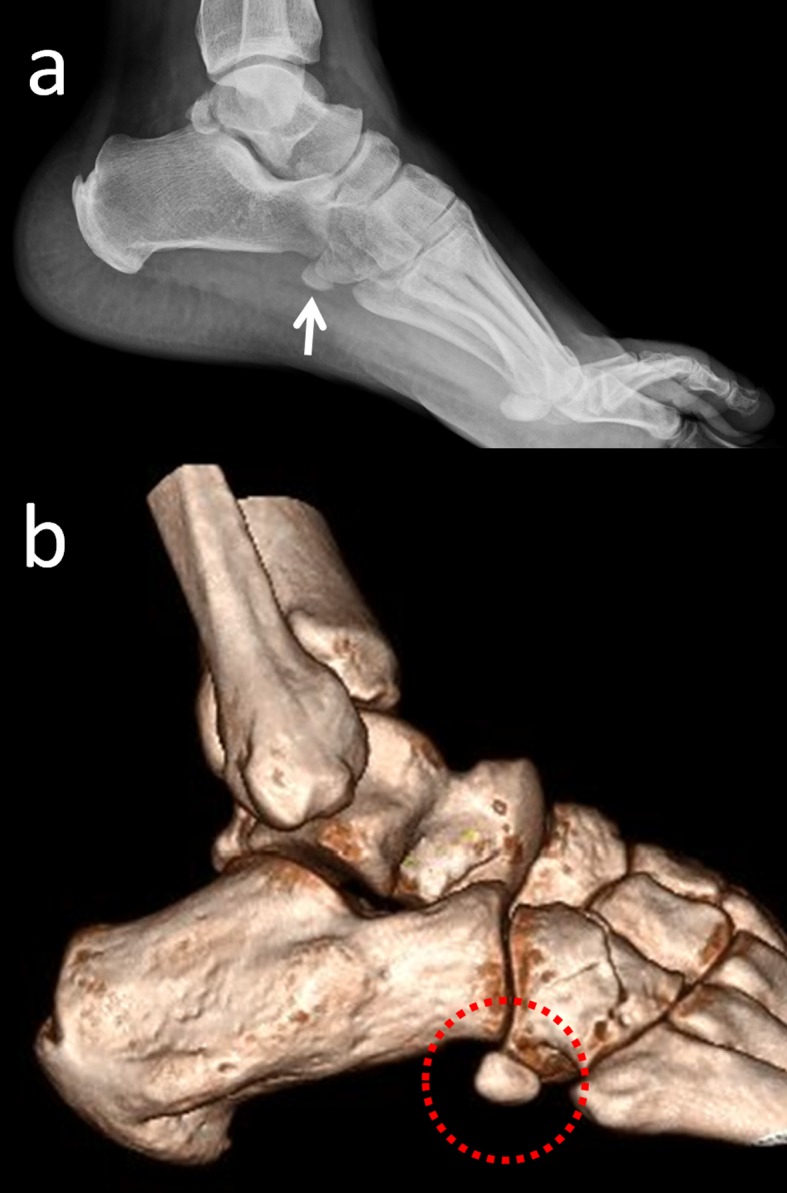
Appearance of os peroneum in a patient with cuboid fracture (a) Lateral foot radiograph; (b) 3D computed tomography (CT) appearance.

Os peroneum is a round or oval-shaped sesamoid bone that is embedded in the peroneus longus tendon. It is located at the lateral plantar aspect of the cuboid and has a reported prevalence of 4.7%-30% (Figure 3) [9,13,18]. 

It can be easily misinterpreted as an avulsion fracture [3,5,9]. On radiographs, it is best seen in an oblique view of the foot. It is bipartite in approximately 30% of cases and bilateral in approximately 60% [5]. Os peroneum syndrome can cause lateral foot pain, tenderness, and swelling along the peroneus longus tendon as well as lateral pain with restricted plantar flexion of the foot. Displacement or fracture of the os peroneum can be indicative of a tear in the peroneus longus tendon [18]. There are different cases of os peroneum such as degenerative arthritis of the pseudo-articulation between the os peroneum and the cuboid, degenerative change between the os peroneum and the cuboid, osteonecrosis of the os peroneum, and aseptic osteonecrosis [5,19].

**Figure 4:**
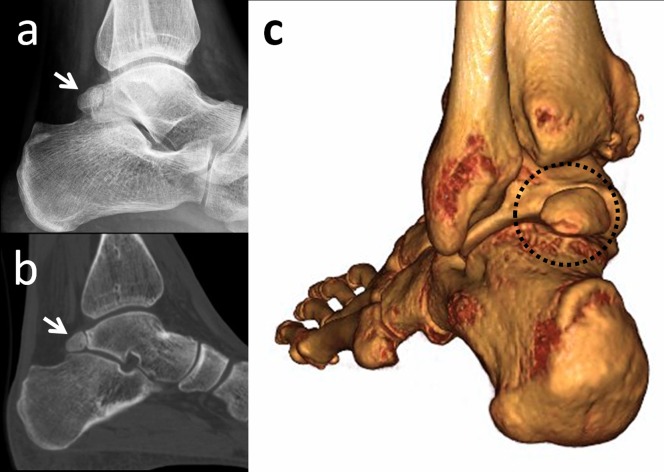
An os trigonum is best seen on lateral foot radiographs (a) Lateral foot radiograph; (b) 2D computed tomography (CT) appearance; (c) 3D CT.

Os trigonum is one of the most common accessory ossicles in the ankle region and usually presents as an incidental radiographic finding. It is located in the posterolateral aspect of the talus and rarely, may be bipartite (Figure 4) [9,20].

The prevalence of this ossicle varies between 1%-25%. Os trigonum may be responsible for chronic ankle pain in children, adolescents, and adults. The most common pathology of the talus and os trigonum is subchondral edema along the synchondrosis [20]. Oestreich reported that a large os trigonum may be associated with flexor hallucis longus tenosynovitis or entrapment [21]. He reported a mega os trigonum and revealed that large and early ossification of an os trigonum helps in the differentiation of progressive pseudo-rheumatoid dysplasia from juvenile rheumatoid arthritis. Richards, et al. presented a study in which athletes were successfully treated with arthroscopic os trigonum resection using posteromedial and posterolateral portals [22].

**Figure 5:**
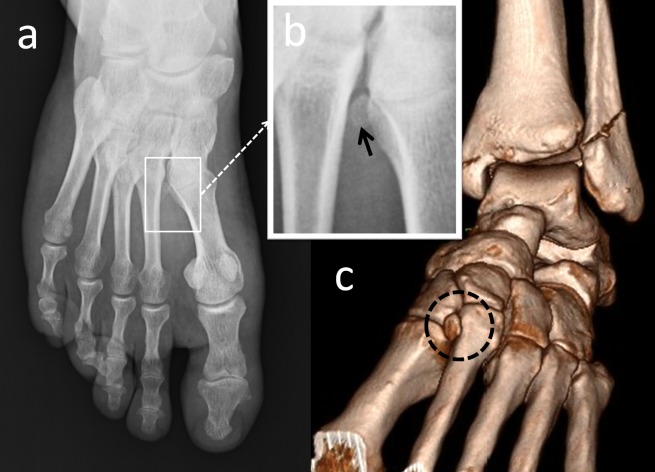
An os intermetatarseum (a) Anteroposterior foot radiograph; (b) Magnified view of the intermetatarsal space, the black arrow is os intermetatarseum; (c) 3D computed tomography (CT) appearance.

Os intermetatarseum is found between the medial cuneiform and the base of the first and second metatarsals (Figure 5).

The os intermetatarseum is less common than the os tibiale externum, os trigonum, and os peroneum. The estimated prevalence is 1.2%-10% [2,9]. Reichmister, et al. reported three cases of painful os intermetatarseum, and described compression of the deep peroneal nerve by the os intermetatarseum [23]. Repeated impact on the instep when kicking the ball might have led to a minor trauma of the deep peroneal nerve above the os intermetatarseum [24]. The os intermetatarseum should be differentiated from fractures of the base of the second metatarsal, which often occur in Lisfranc dislocations. If there is no fracture site, no soft tissue swelling, and no mechanism of injury, Lisfranc dislocation can be ruled out. In addition, os intermetatarseum may be seen together with hallux valgus deformity and should be differentiated from a calcified dorsalis pedis artery. When evaluating dorsal midfoot pain, os intermetatarseum should be taken into consideration.

**Figure 6:**
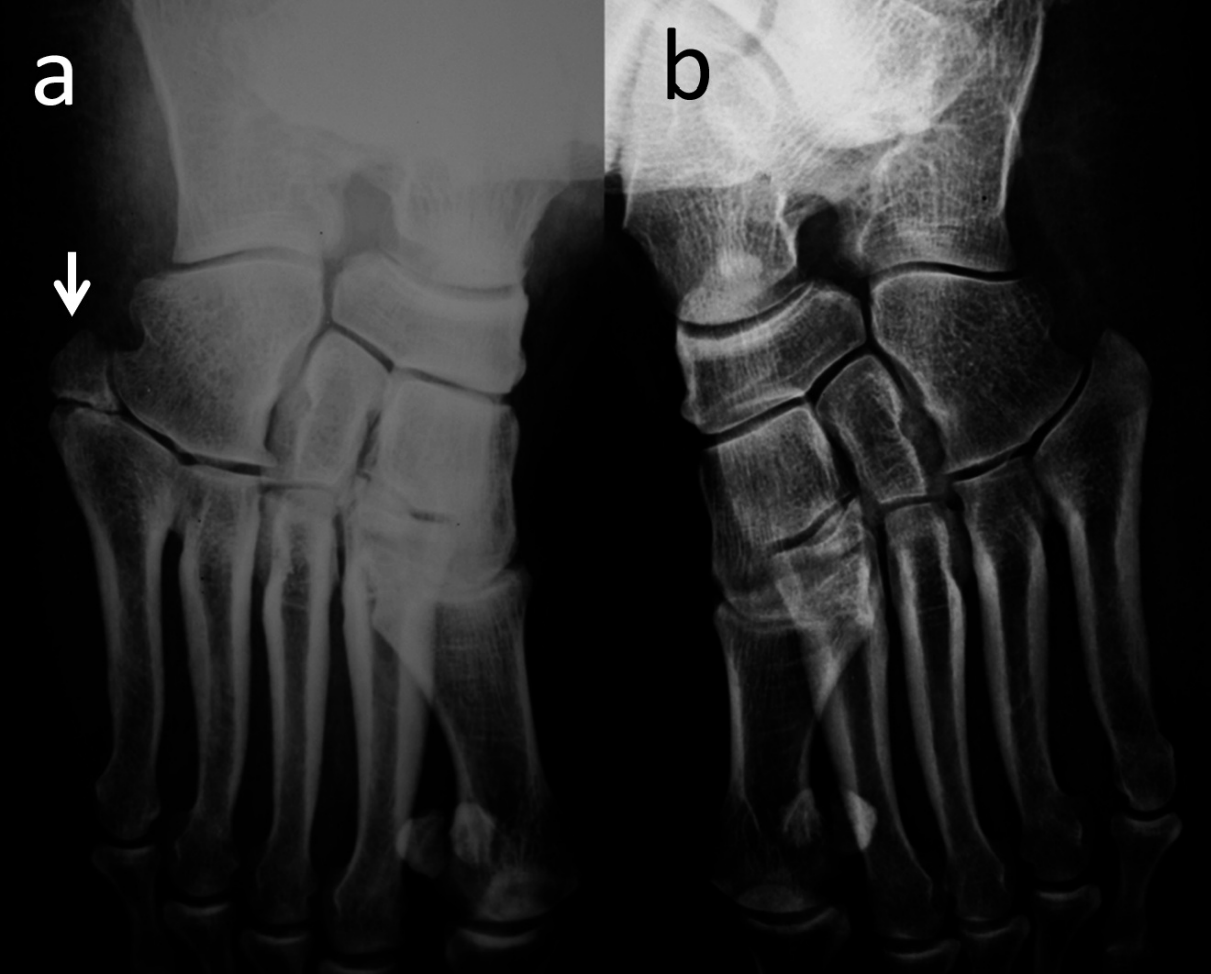
Bilateral anteroposterior foot radiograph of a patient with unilateral os vesalianum pedis (a) Right foot (white arrow) is os vesalianum pedis; (b) Normal left foot.

Os vesalianum pedis is a small accessory ossicle adjacent to the tip of the well-developed tuberosity of the fifth metatarsal (Figure 6).

It is a very rare accessory bone, with 0.1%-5.9% prevalence. A lateral oblique radiograph is the best method for demonstrating accessory ossicles and its articulations. It is mostly asymptomatic and incidentally recognized, but may sometimes cause lateral foot pain. The major objective in diagnosis is to differentiate os vesalianum pedis from acute avulsion fractures of the fifth metatarsal, Jones fracture, Iselin’s disease, stress fractures, non-union of the fifth metatarsal base, non-united apophysis of the fifth metatarsal base, and os peroneum. Clinically, there is edema, tenderness, and sometimes ecyhmosis around the base of the fifth metatarsal. In avulsion fractures, the fragment is a sharply delineated piece of bone that lacks cortication at the fracture line. Conversely, os vesalianum pedis is surrounded by a bony cortex and the margins are rounded [25].

**Figure 7:**
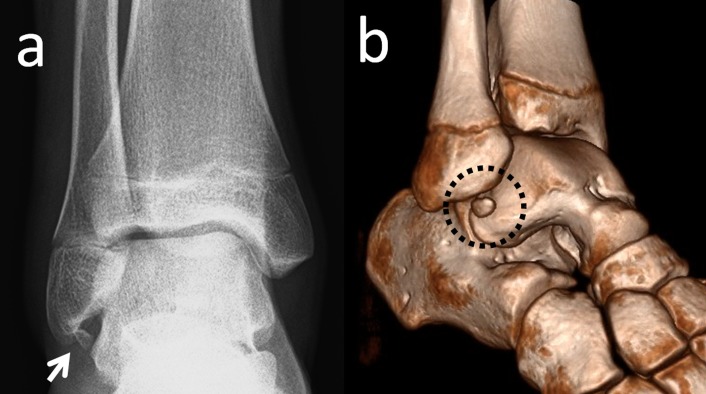
An os subfibulare (a) Anteroposterior foot radiograph, the white arrow is os subfibulare (b) 3D computed tomography (CT) appearance.

Os subfibulare is located beneath the lateral malleolus. It is a round or comma-shaped bone which is rarely seen (Figure 7).

**Figure 8:**
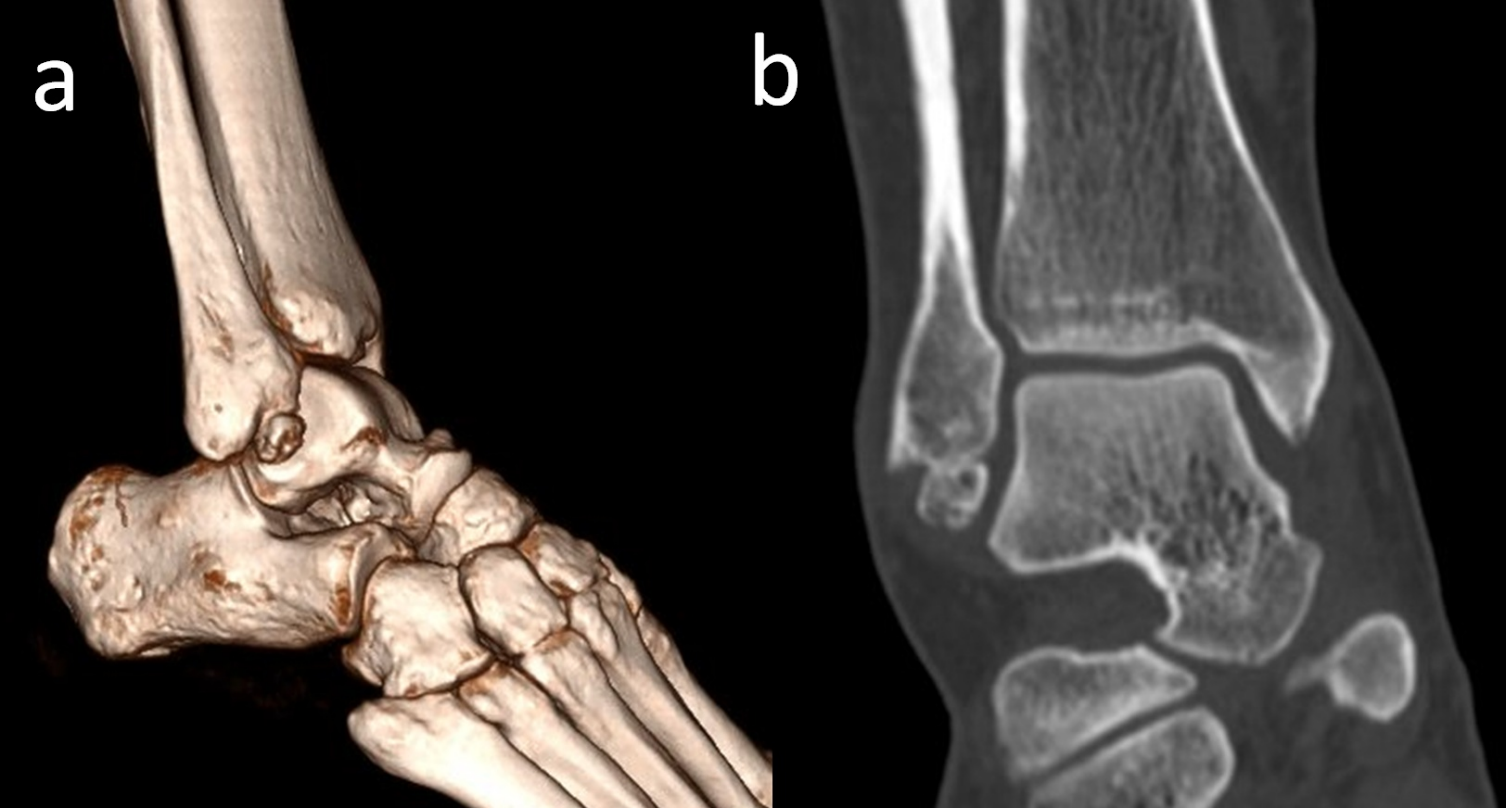
A 42-year-old male patient with a united anterior talofibular ligament avulsion fracture (a) Union is seen on coronal computed tomography (CT); (b) 2D CT appearance.

It might be 5-10 mm in size and is seen quite easily on anteroposterior radiographs of the ankle joint. The incidence of os subfibulare has been reported between 0.2%-6.6% in the literature [13]. There are conflicting opinions whether the os subfibulare is a true accessory ossicle or an old non-united avulsion fracture of the anterior talofibular ligament (Figure 8) [26-29]. 

**Figure 9:**
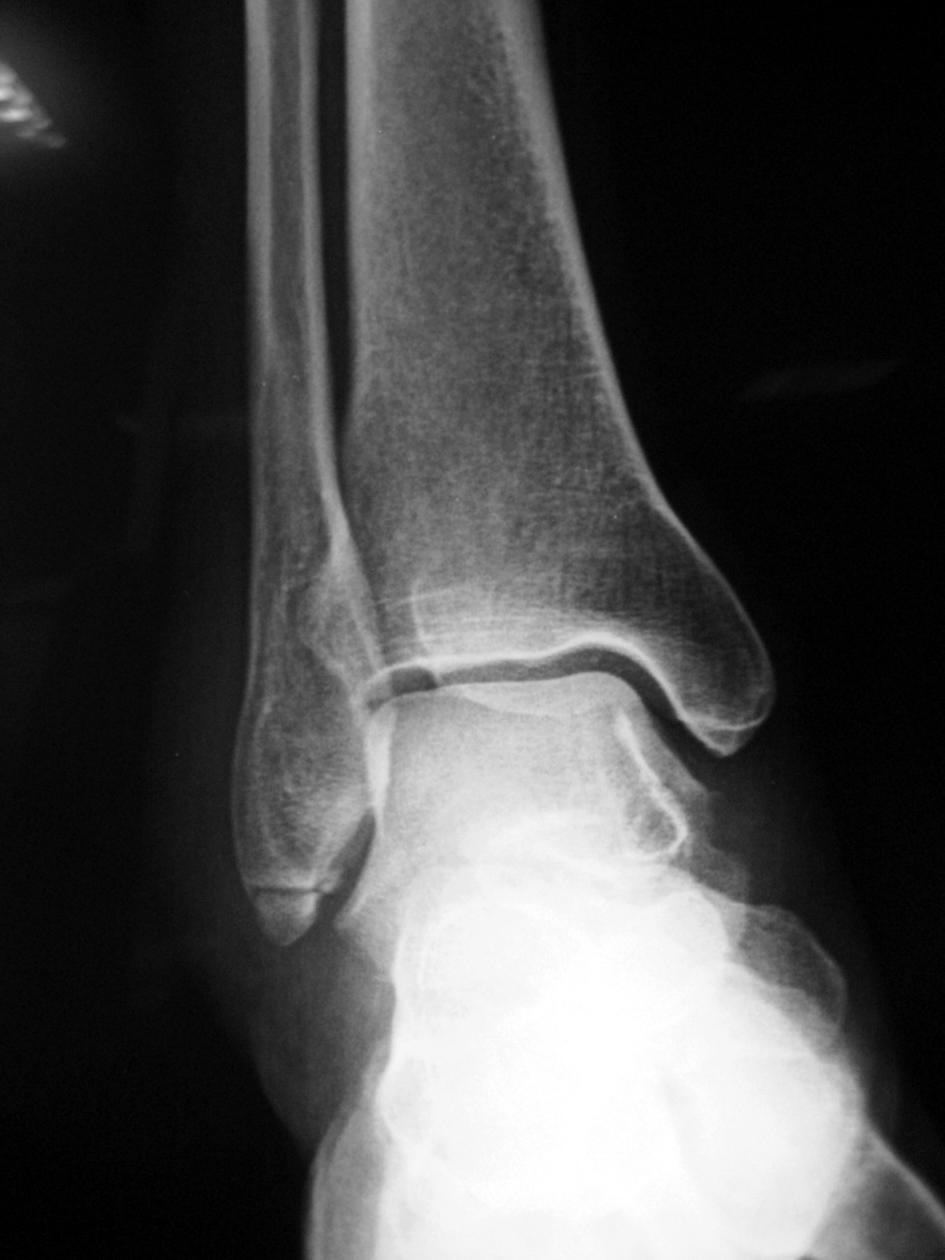
A patient with an avulsion fracture of the tip of the lateral malleolus Please note the similarity between an avulsion and a true ossicle.

Os subfibulare could be confused with an acute avulsion fracture of the lateral malleolus (Figure 9). 

As the pain, swelling, and localization of the tenderness are similar, distinguishing an acute ankle sprain from a fracture might be difficult. Although differentiation is not easy, radiographic findings which are peculiar to os subfibulare may help. Tenderness over the distal fibula, a missing part of the lateral malleolus, and a sharp uncorticated fracture line without sclerosis are important findings for acute avulsion fractures [10]. Pill, et al. suggested that in children with chronic pain and instability associated with an os subfibulare, surgical excision of the os subfibulare combined with reconstruction of the anterior talofibular ligament was effective in restoring ankle stability [30].

**Figure 10:**
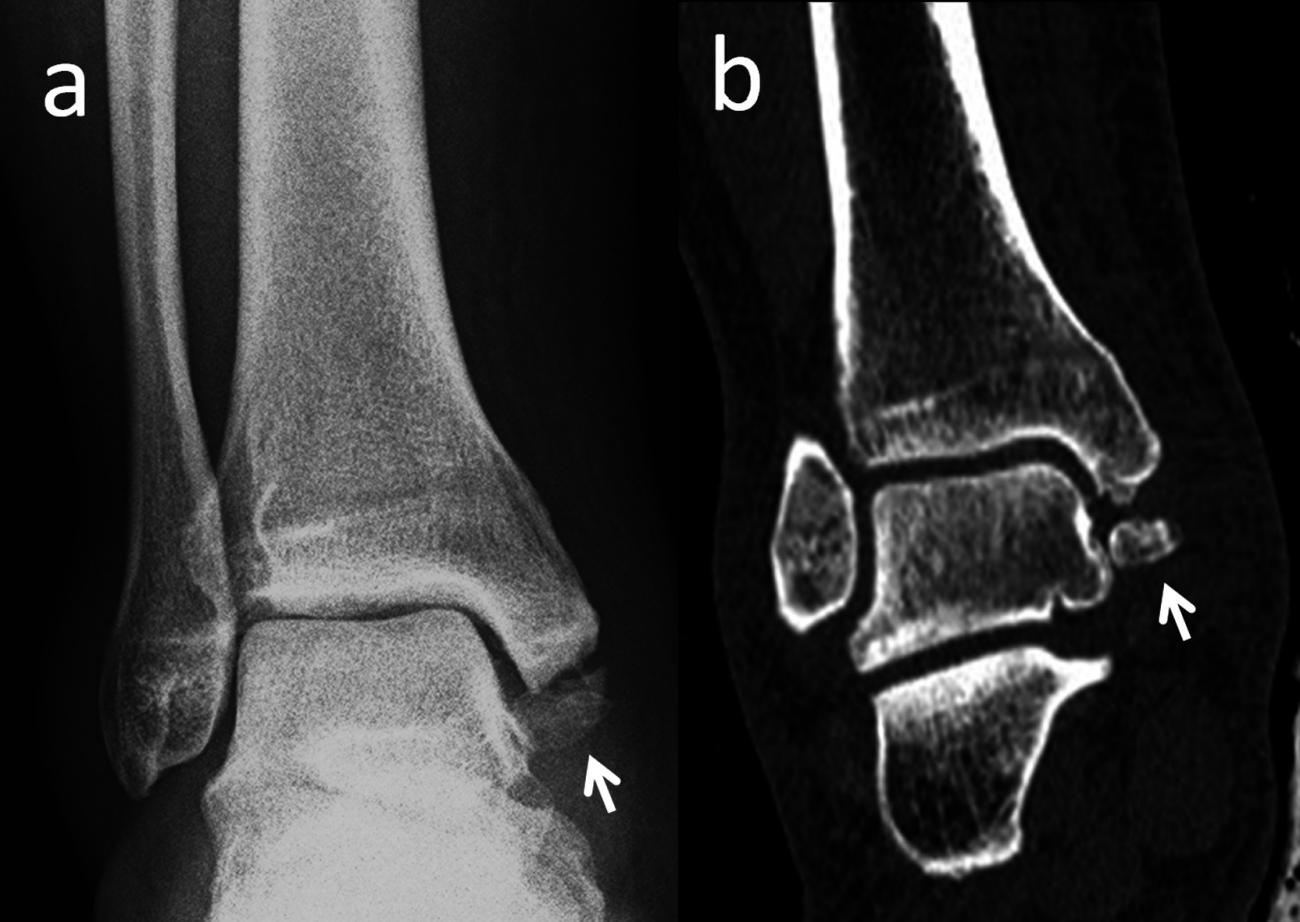
A 36-year-old patient with an os subtibiale Note the sclerotic edges of the ossicle and completely normal appearance of the medial malleolus.

Os subtibiale is located at the posterior aspect of the medial malleolus (Figure 10).

It is a rare incidental accessory bone, 4-15 mm in size with an estimated prevalence of 0.7%-1.2% [31]. Os subtibiale might seem like an abnormal ossification in ankle radiographs and it could be confused with accessory ossification centers, posttraumatic ossification, or avulsion fractures [32].The differentiation of an ossubtibiale from an acute fracture is the major diagnostic goal, although it may be necessary to differentiate it from an unfused ossification center. The relationship to an anterior or posterior colliculus may help to differentiate an unfused ossification center from an os subtibiale [11]. When a patient presents with ankle trauma, there are some features that help differentiate the os subtibiale from a fracture. First, the edges of the accessory ossicles are smooth and rounded on the radiographs but a fractured malleolus is mostly irregular. Secondly, the os subtibiale is often bilateral and is present just below the region of the medial malleolus [31]. Surgical excision of this bone is rarely necessary. Even in the case of acute trauma overlying such an accessory bone, conservative treatment is usually adequate [2]. In addition to the confusion with acute fractures, os subtibiale could also lead to posterior tibial tendon dysfunction [33].

**Figure 11:**
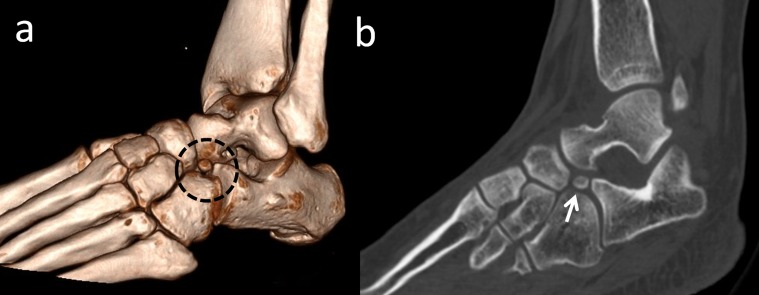
Os calcanei secundarium (a) 3D computed tomography (CT) appearance in the black dotted circle; (b) 2D CT appearance, the white arrow is os calcanei secundarium.

Os calcanei secundarium is located dorsal to the calcaneus in an interval between the anteromedial aspect of the os calcaneus, the proximal aspect of the cuboid and navicular, and the head of the talus (Figure 11). 

**Figure 12:**
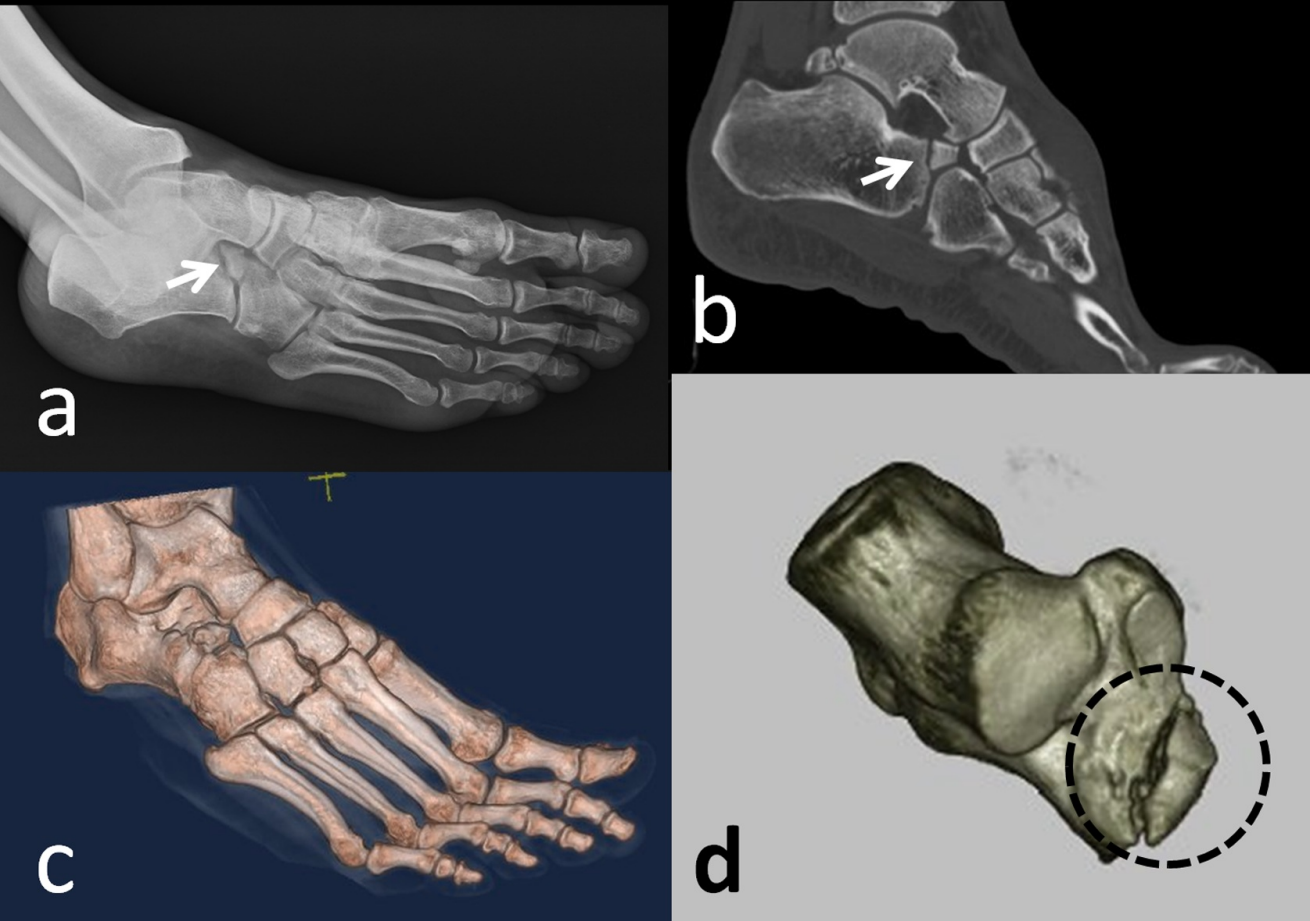
A patient with an anterior process fracture of the calcaneus (a) Foot radiograph; (b) 2D computed tomography (CT) appearance; (c) 3D appearance; (d) 3D appearance - note the sharp edges, communition, and jigsaw puzzle relationship between the fragment and the calcaneus.

Moreover, it may form a set of articulations with the cuboid and the talus. It is a rare accessory ossicle with a 0.4%-11% incidence [2]. It may be round or triangular shaped. It is a large-sized accessory bone (8/12/22 mm) and is visible on a lateral oblique radiograph of the hindfoot [34]. Sometimes, patients may complain of restricted subtalar motion and pain. Wagner, et al. reported excision of the anterior tuberosity of the calcaneus for treatment of chronic pain [35]. Calcaneus secundarius should be differentiated from an anterior process fracture of the calcaneus, fracture of the tuberosity of the calcaneus, calcaneus accessorius, cuboideum secundarium, and os sustentaculi (Figure 12).

Os calcanei accessorium (os trochleare, os talocalcaneale laterale) is located near the trochlear process of the calcaneus on the fibular aspect just distal to the fibular malleolus. This bone is approximately 5 mm and can be seen on a dorsoplantar radiograph of the foot. The calcaneus accessorius should be differentiated from an os subfibulare or an avulsion fracture [36]. 

**Figure 13:**
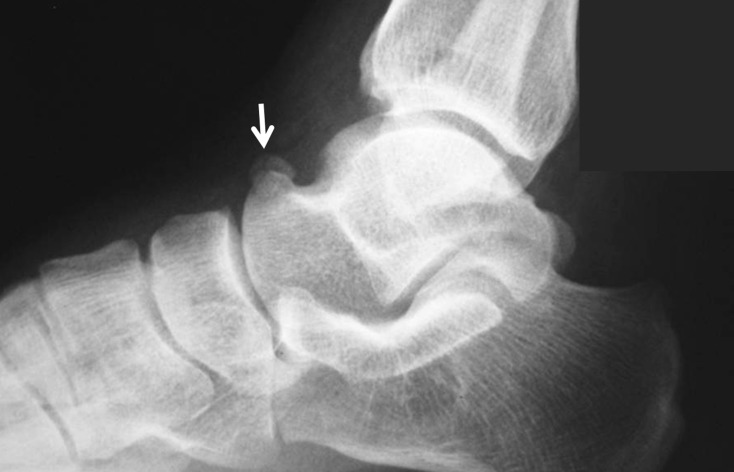
Radiographic appearance of the os supratalare located over the head of the talus (white arrow)

Os supratalare is located on the dorsum of the talus between the ankle and the talonavicular joint and typically over the ridge along the talar head/neck, but may also be seen distally over the head (Figure 13).

It may be fused with the talus or remain as a free accessory bone. It is a rare skeletal variant up to 4 mm in size and 0.2%-2.4% prevalence. It can easily simulate an old, non-united avulsion fracture and can only be identified in the lateral view. A few studies focusing on os supratalare have been published [2]. Although os supratalare is usually asymptomatic, Kim, et al. reported a symptomatic case with a hard bump and dorsal hind foot pain [37]. It can also cause pain or degenerative changes in response to over use and trauma.

Os sustentaculi is located on the posterior aspect of the sustentaculum tali with 0%.3-1% prevalence and it can be seen on anteroposterior radiographs and lateral projection view of the hindfoot. This bone may be joined to an accessory joint between the sustentaculum tali and the talus. Os sustentaculi is a rare entity, which may be depicted on axial or coronal MRI, and could be confused with a bone tumor, fracture, or an exostosis. Surgical excision is rarely required [38,39].

**Figure 14:**
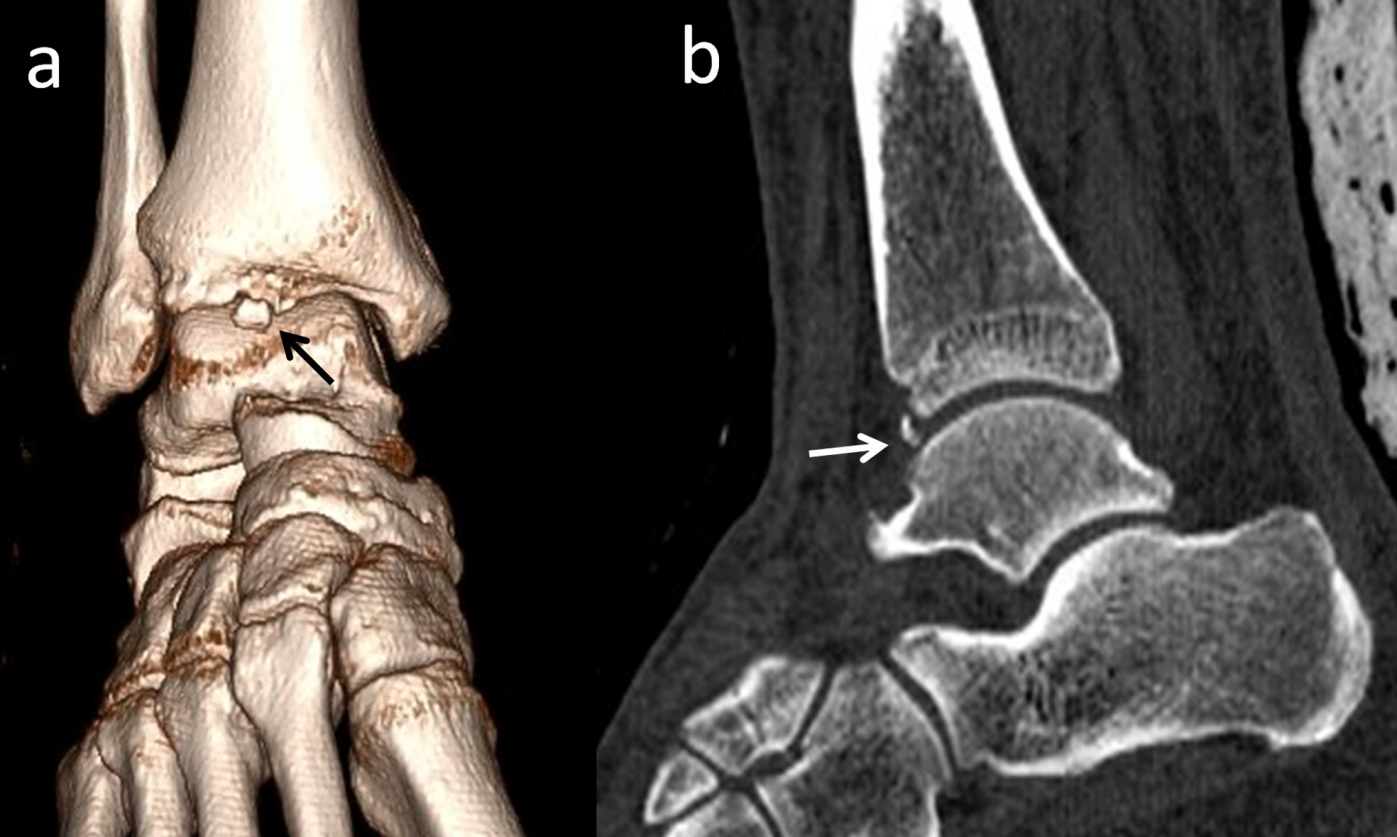
A patient with anterior ankle impingement syndrome (a) 3D computed tomography (CT) appearance, the black arrow shows the os talotibiale; (b) 2D CT appearance, the white arrow shows the os talotibiale.

Os talotibiale is a very rare ossicle located anterior to the tibiotalar joint with a 0.5% incidence. However, there is insufficient knowledge in the literature and no available published case reports about this bone. This bone may cause anterior ankle impingement syndrome (Figure 14) [13].

Os tali accessorium and os talus secundarius are often confused with each other. The os tali accessorium, with an incidence of 0.02%, is situated beside the trochlea tali [13]. The talus accessorius is usually on the medial side of the talus and may form in the deltoid ligament. Talus accessorius is similar to the intercalary sesamoid bone between the external malleolus and the talus. To the best of our knowledge, there are no case reports about these bones in the literature. Os talus secundarius is characteristically located along the lateral aspect of the talus. It is a very rare accessory bone with a 0.1 % prevalence [13]. Oliveria, et al. reported that if this ossicle is large, it limits the subtalar range of motion, causing pain and restriction mainly during sportive activities and during intense daily life activities. They suggested that the current standard treatment is surgical excision which will provide pain relief and a greater range of motion [40]. Viana, et al. stated that CT and MRI may be useful in its diagnosis, characterization, and in operative planning [41]. Differential diagnosis between talus secundarius and talus accessorius can usually be made radiographically. It may be confused with the more common os subfibulare or os tali accessorium, but the latter is located on the medial side of the talus and the former represents an accessory ossification centre just under the tip of the lateral malleolus [40].

Os subcalcis is found on the plantar aspect of the calcaneus slightly posterior to the insertion of the plantar fascia. This bone may have a reach of up to 10 mm in diameter. Knowledge of this bone is insufficient because there are no reported cases in the literature [2].

Os cuboideum secundarium is a rare accessory ossicle that is located adjacent to the cuboid and calcaneus. The exact prevalence is unknown, as there are only a few case reports in the literature. It has been reported that surgical removal of this bone in a child is successful in the alleviation of pain [42]. In two reported cases, the bone was 'free' and situated adjacent to the cuboid and calcaneus without osseous coalition to any neighboring bone. In another case, the bone was associated with scalloping of the inferior margins of the calcaneus and cuboid bones at their articulation, without the involvement of the navicular. Knowledge of this rare accessory ossicle, together with the foresight to obtain radiographs before MRI, could prevent this type of confusions [43].

**Figure 15:**
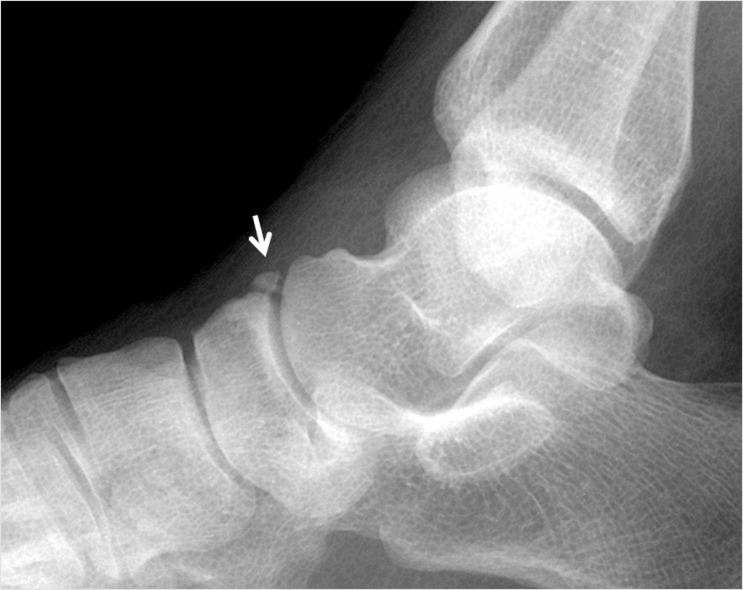
Lateral radiograph of the foot that demonstrates os supranaviculare (white arrow)

Os supranaviculare, also known as os talonaviculare dorsale, talonavicular ossicle, and Pirie’s bone, is located on the dorsal aspect of the talonavicular joint, close to the midpoint (Figure 15).

**Figure 16:**
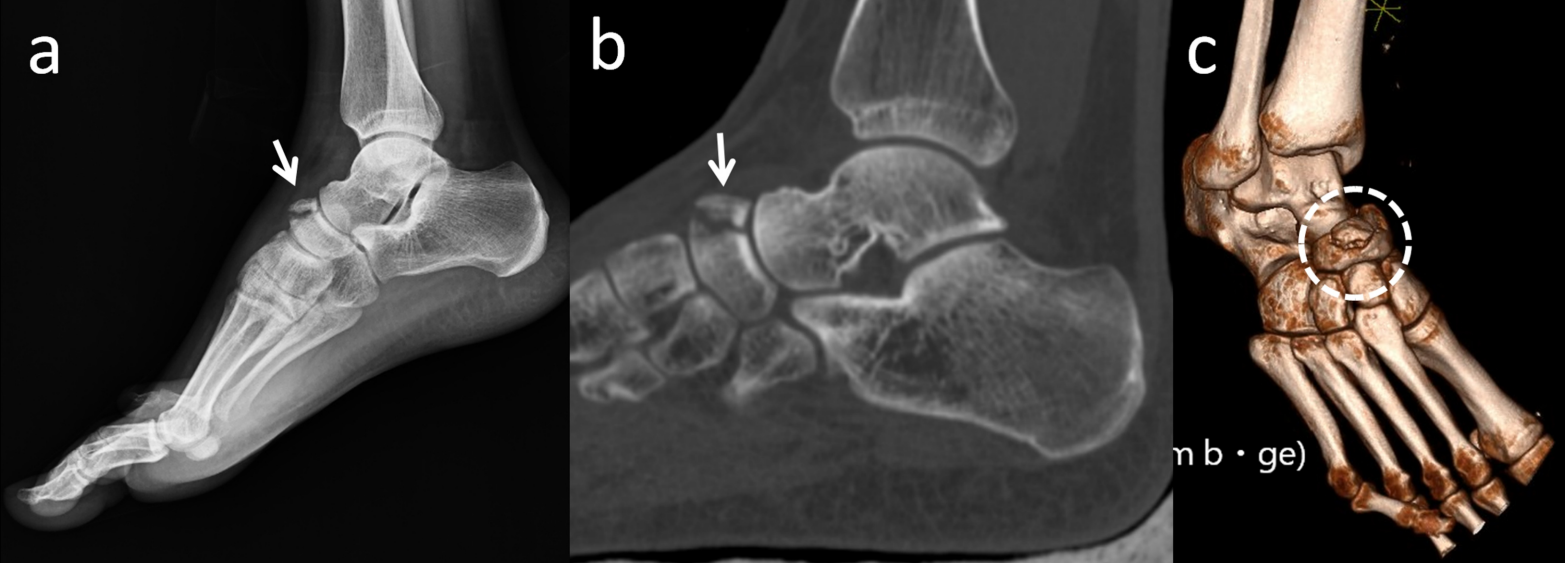
A 51-year-old female patient with an avulsion fracture of the navicular bone (a) Foot radiograph; (b) Sagittal 2D appearance (c) 3D computed tomography (CT) of the fracture - note the similarity between an os supranaviculare and an avulsion fracture.

Its prevalence has been reported as 1%-3.5 % [13,24,44]. The ossicle may be fused with the talus or with the navicular bone [2]. Pavlov, et al. reported a series of 23 navicular stress fractures, 22% of which involved an os supranaviculare [13,19]. The exact cause of this association is not clear, but it is possible that a pre-existing dorsal cortical notch accompanying an os supranaviculare may contribute to it. Avulsion fractures can be differentiated from os supranaviculare, os supratalare, or os talotibiale by its irregular surface and lack of cortication, and by the patient’s history of trauma (Figure 16) [19,45].

In addition, osteoarthritic degeneration of the talonavicular joint should be differentiated from this accessory bone. Miller and Black reported a case with impingement of the deep peroneal nerve because of an os supranaviculare [44]. During operations of these bones, the deep peroneal nerve, and dorsalis pedis artery and vein must be protected.

Os infranaviculare (cuneonavicular ossicle) is situated dorsally between the navicular and the first cuneiform, usually overriding the latter [46]. The literature about this bone is restricted. Sizensky and Marks suggested that it might be confused with avulsion fractures, degenerative arthrosis of the perinavicular joints, or Mueller-Weiss syndrome, but, if the morphology of the navicular bone is normal, it can help in the differentiation of these accessory ossicles from the other cases [47]. Kim and Roh described a case with painful os infranaviculare; advanced degenerative change between the navicular and accessory bone was considered as the main cause of the foot pain [48].

Os paracuneiforme is an ossicle lying in a hollow on the medial side of the foot between the navicular and medial cuneiform bones. The incidence of this bone is unclear. Although it is always included in the lists of accessory ossicles, there have been few reported cases of this bone. Most of the descriptions are based on radiographic findings. Morrison described a case with os paracuneiforme where surgery was required because of bony swelling accompanied by foot pain [49]. It was emphasized that although such bones are rarely seen, they could cause symptoms requiring surgical intervention. 

Os intercuneiforme is located in an interval between the first and second cuneiforms just distal to the navicular. It is typically triangular shaped. It is a rare incidental skeletal variant with 1%-2% prevalence. Prescher reported that with rare occurrence and its small dimensions, the os intercuneiforme is not expected to be of any practical or clinical significance [50].

Os cuneometatarsale I tibiale is an accessory bone which occurs near the medial aspect of the first metatarsocuneiform joint. This bone should be differentiated from the os paracuneiforme and the accessory navicular bone [2].

Os cuneo-I metatarsale-I plantare occurs on the plantar aspect of the first metatarsocuneiform joint. It articulates with the plantar base of the first metatarsal and the first cuneiform. It is not often seen on a dorsoplantar radiograph but may be seen on a lateral oblique view [2].

Os cuneo-I metatarsale-II dorsale lies on the dorsal aspect of the articulation of the second metatarsal and second cuneiform. It is wedge-shaped with the base oriented dorsally and characterized as 'pepper corn' in size. This is also difficult to identify on a radiograph but may be seen on a lateral or lateral oblique view [2]. There is insufficient information in the literature about the three above-mentioned bones.

Os aponeurosis plantaris lies enclosed in the plantar aponeurosis and may vary significantly in size. It is usually oblong and flat and best seen on a lateral radiograph. It should be differentiated from a calcaneal spur or a fracture of the calcaneal spur [2]. However, knowledge about this bone is inadequate.

## Conclusions

In the present paper, all accessory ossicles defined in the available literature have been reviewed. Understanding the possible disorders of these ossicles can provide a more accurate diagnostic process. Diagnosing and reporting might be helpful to find real incidence and clinical importance of these bones. This article describes the normal anatomy, morphological variations, abnormal variations, differential diagnosis, and the clinical importance of the accessory ossicles as well as the management of pathological conditions. In this context, this review can be considered a good guide for physicians in this field.
